# Disrupted social memory ensembles in the ventral hippocampus underlie social amnesia in autism-associated Shank3 mutant mice

**DOI:** 10.1038/s41380-021-01430-5

**Published:** 2022-02-04

**Authors:** Kentaro Tao, Myung Chung, Akiyuki Watarai, Ziyan Huang, Mu-Yun Wang, Teruhiro Okuyama

**Affiliations:** 1 Laboratory of Behavioral Neuroscience, Institute for Quantitative Biosciences (IQB), The University of Tokyo, Tokyo, 113-0032 Japan; 2 PRESTO, Japan Science and Technology Agency (JST), Kawaguchi, Saitama 332-0012 Japan

**Keywords:** Neuroscience, Autism spectrum disorders

## Abstract

The ability to remember conspecifics is critical for adaptive cognitive functioning and social communication, and impairments of this ability are hallmarks of autism spectrum disorders (ASDs). Although hippocampal ventral CA1 (vCA1) neurons are known to store social memories, how their activities are coordinated remains unclear. Here we show that vCA1 social memory neurons, characterized by enhanced activity in response to memorized individuals, were preferentially reactivated during sharp-wave ripples (SPW-Rs). Spike sequences of these social replays reflected the temporal orders of neuronal activities within theta cycles during social experiences. In ASD model *Shank3* knockout mice, the proportion of social memory neurons was reduced, and neuronal ensemble spike sequences during SPW-Rs were disrupted, which correlated with impaired discriminatory social behavior. These results suggest that SPW-R-mediated sequential reactivation of neuronal ensembles is a canonical mechanism for coordinating hippocampus-dependent social memories and its disruption underlie the pathophysiology of social memory defects associated with ASD.

## Introduction

The ability to recognize and memorize familiar conspecifics is crucial for animals that engage in social interactions [1, 2]. Several brain regions have been identified as being associated with the experience-dependent encoding of social characteristics in rodents [3, 4]. In particular, the ventral CA1 sub-region of the hippocampus (vCA1) contains neurons that respond to other individuals [5] but not to inanimate objects [6] and has been identified as a key locus for social memory storage [5, 7]. In vivo Ca^2+^ imaging in freely moving mice revealed that a subset of vCA1 neurons is activated by the presence of familiar mice during social interactions [5]; however, how the activities of these social neurons are organized and maintained at the fine temporal resolution necessary for effective memory encoding and recall remains unknown.

Hippocampal sharp-wave ripples (SPW-Rs) are transient, high-frequency, field oscillations that are typically observed during slow-wave sleep and quiet wakefulness [8] and play a pivotal role in the formation of episodic memories encoded in the hippocampus. In the dorsal hippocampus, place-cell ensembles that are activated during awake exploration are sequentially reactivated during SPW-Rs that occur during both slow-wave sleep [9–11] and transient immobility during awake exploration [12, 13], which have been suggested to play prominent roles in synaptic plasticity [14] and memory consolidation [15, 16]. SPW-Rs can also be observed in the ventral hippocampus [17], where they are thought to distribute behavior-contingent information to multiple downstream regions [18]. However, the SPW-Rs that arise in the ventral segment of the hippocampus often remain isolated [17]. To date, it remains unclear whether the ventral hippocampus neuronal activity during SPW-Rs inherently represents spatial information or is used for processing related to other aspects, such as social information.

Impairments in social reciprocity result in debilitating symptoms associated with several neurodevelopmental disorders, including autism spectrum disorders (ASDs) [19, 20]. SH3 and multiple ankyrin repeat domains 3 (*Shank3*), one of the most promising ASD-susceptibility genes [21], encodes a postsynaptic scaffolding protein [22], and ASD model mice generated by deleting *Shank3* are characterized by reduced social interactions and abnormal social novelty recognition [23, 24]. Although social memory impairment is a hallmark of ASD both in human patients [25] and in mouse models [26], it remains elusive whether any alteration in social information processing in the hippocampus underlies such impairments. Here, we report that vCA1 neurons that respond to familiar social targets are preferentially co-activated during SPW-Rs following social memory formation. The relative spike timing of these social neurons was preserved across SPW-Rs, indicating that social information is likely organized based on a temporal code. Furthermore, in *Shank3*-knockout (KO) mice, a genetic model of ASD, the proportion of social memory neurons was reduced, and vCA1 neurons exhibited a loss of temporal sequence consistency during SPW-Rs that was correlated with impaired social discrimination behavior. These results indicate that social information is processed in the ventral hippocampus through a mechanism that is analogous to that used to process spatial information in the dorsal hippocampus and that this mechanism for social information processing is impaired in a genetic model of ASD.

## Materials and methods

### Mice

All procedures were in accordance with protocols approved by the Institutional Animal Care and Use Committee at the Institute for Quantitative Biosciences, University of Tokyo. All animals were socially housed under 12 h (7 a.m.–7 p.m.) light/dark cycle conditions, with ad libitum access to food and water. C57BL/6J wild-type (WT) mice were obtained from the Central Laboratories for Experimental Animals, Japan, and *Shank3*-KO (KO) mice were obtained from the Jackson laboratory (B6.129-Shank3^tm2Gfng^/J, Stock No: 017688). The *Shank3*-KO allele has a neomycin cassette replacing the PDZ domain (exons 13–16) of the *Shank3* gene. Ten adult male C57BL/6J mice (28–32 g, 3–4 months old) and ten adult male homozygous *Shank3*-KO mice (26–32 g, 3–4 months old) were used as subject mice. Young adult male C3H and BALB/c mice (22–28 g, 6–8 weeks old) were used as stimulator mice.

### In vivo electrophysiology

During the SDT and the subsequent resting period, mice were connected to a 64-channel amplifier board (RHD2164; Intan Technologies, Los Angeles, CA), and neural activities were sampled at 30 kHz using the Open Ephys data acquisition system [27]. The wide-band signal was downsampled to 1.25 kHz and used as the local field potential (LFP) signal. Spike sorting was performed automatically using Kilosort2 [28]. This was followed by manual adjustment of the waveform clusters using the phy software [29]. Units with firing rate less than 15 Hz and trough-to-peak length of spike shapes longer than 0.5 ms were classified as excitatory neurons [30, 31].

### Behavioral analysis

Positions of the center of the body and the base of the tail of subject mice were detected using DeepLabCut [32]. The nose of subject mice were frequently occluded by the head-mounted apparatus and its detection was unstable; therefore, we excluded it from the analysis. The relative distance (*d*) between the subject mouse and two stimulator mice in each frame was calculated as (*d*_*A*_ − *d*_*B*_)/(*d*_*A*_+*d*_*B*_), where *d*_*A*_ and *d*_*B*_ are the distances between the body position of the subject and the corners of the social arena where stimulator mouse A or B, respectively, were placed. Coordinates with |*d*| ≥ 0.5 (corresponding approximately to twice the social chamber’s radius) were defined as being in the social zone. Discrimination score of the subject mouse was computed as (*Dur*_*A*_ − *Dur*_*B*_)/(*Dur*_*A*_ + *Dur*_*B*_), where *Dur*_*A*_ and *Dur*_*B*_ are the total time spent in the social zone in a comprehensive period of 10 min of social trials around the stimulator mouse A or B, respectively. The subject’s egocentric direction relative to each stimulator mouse was calculated by the relative orientation of the tail-to-body vector of the subject toward the corners of the arena where stimulator mice were placed.

### Unit coactivation analysis

To measure the pairwise coactivation of units during online recordings, we used the cosine similarity between two vectors of spike counts, calculated as:

$$Similarity = \frac{{\mathop {\sum }\nolimits_k {{{{{{{\mathbf{U}}}}}}}}_i(k) \cdot {{{{{{{\mathbf{U}}}}}}}}_j(k)}}{{\sqrt {\mathop {\sum }\nolimits_k {{{{{{{\mathbf{U}}}}}}}}_i(k)^2} \sqrt {\mathop {\sum }\nolimits_k {{{{{{{\mathbf{U}}}}}}}}_j(k)^2} }}$$

where **U**_*i*_*(k)* and **U**_*j*_*(k)* are spike counts of unit *U*_*i*_ and *U*_*j*_ within the *k*th movie frame. Likewise, the pairwise coactivation of units during ripple events was evaluated using the same formula but with **U**_*i*_*(k)* and **U**_*j*_*(k)* being the spike counts of unit *U*_*i*_ and *U*_*j*_ during the *k*th ripple event. For offline recordings, the significance of the calculated similarity was determined by a permutation test of spike counts during each ripple event (repeated 100,000 times). The *P* value represents the proportion of shuffled values larger than the actual observed value.

### Spike sequence analysis

The LFP signal was bandpass filtered (6–12 Hz) and averaged across channels having pyramidal cells. Theta cycles started at the peak of the LFP. Cycles shorter than 80 ms or longer than 200 ms were discarded, and only periods containing at least three contiguous cycles with both troughs and peaks larger than 1 SD were selected for further analysis. Awake ripples were excluded from the analysis by detecting ripples using the same criteria for offline recordings but with no constraint by the multi-unit activity (MUA). Rank order of a neuron in each online theta cycle or offline ripple event was defined as the normalized temporal position of a first spike in the sequence of all cells that participated in that theta cycle or ripple event. Delta rank order was determined as the difference of mean rank orders between two cells. The orders of the cells were determined so that the differences during theta cycles were always a positive value.

### Statistics and general methods

No statistical methods were used to predetermine the sample size, which was similar to those reported in previous publications [5, 6, 33]. All analyses were performed using MATLAB (MathWorks) unless otherwise noted. All data are expressed as the mean ± standard error of the mean unless otherwise noted. Data collection and analysis were not performed blind because the experimental conditions were obvious to the researchers. All behavioral experiments were controlled by computer systems, and data were collected and analyzed in an automated and unbiased way. Sessions were excluded if the number of simultaneously recorded putative pyramidal cells was lower than two, or if the animals did not enter either side of the social zone during at least one trial. In the instances where parametric statistics were performed, data distribution was assumed to be normal, but this was not formally tested. If normality could not be apparently assumed, nonparametric statistics (Wilcoxon signed-rank test or Wilcoxon rank-sum test) were performed. A detailed description of statistics, including individual data points, *P* values and further statistical parameters, are provided in the figure legends. Statistically significant results are indicated in the figures using *P  <  0.05, **P  <  0.01 and ***P  <  0.001.

## Results

### Single-unit recording of vCA1 neurons during a social discrimination test

**Fig. 1:**
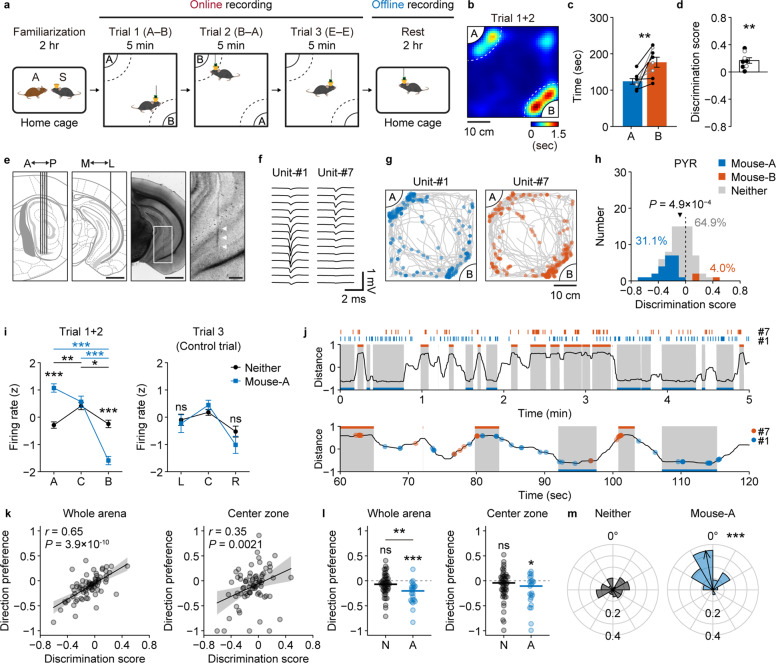
Electrophysiological recordings of ventral CA1 social neurons. **a** Schematic of the behavioral paradigm. After 2 h of familiarization between a subject mouse (S) and a stimulator mouse-A in a home cage, the subject mouse was allowed to explore a social arena where the familiar mouse-A and a novel mouse-B were placed. Dotted lines in the arena indicate the borders of social zones. **b** Heat map of occupancy time during Trial 1 and Trial 2 from a representative session. Note that the map for Trial 2 is rotated and superposed onto that of Trial 1. Comparison of duration spent in the social zone (**c**) and the discrimination score (**d**). *n* = 8 mice; **P = 0.0054, Cohen’s *d* = 1.40, paired *t*-test (**c**) and **P = 0.0078, *r* = 0.94, Wilcoxon signed-rank test (**d**). Data from mice that only underwent SDT are shown as gray lines and blank circles, while data from mice that also underwent electrophysiological recordings from vCA1 are shown as black lines and filled circles. **e** A four-shank silicon probe was implanted in the ventral hippocampus of freely moving mice. Arrowheads in the magnified view indicate the probe track. Scale bars: 1 mm (unmagnified images) and 200 μm (magnified images). **f** Spike waveforms of two representative clusters. Data recorded from 14 channels at the shank tip are shown. **g** Spatial firing patterns units shown in **f**. Unit-#1, a mouse-A cell. Unit-#7, a mouse-B cell. Blue or red dots represent body positions at each spike timing of the cell and gray lines represent the trajectories of the subject mouse during Trials 1 and 2. **h** Discrimination scores of recorded pyramidal neurons (*n* = 75 cells). Blue, mouse-A cells (*n* = 23 cells, 31.1%); red, mouse-B cells (*n* = 3 cells, 4.0%); gray, neither neurons (*n* = 48 cells, 64.9%). The *P* value indicates that the median value of all pyramidal neurons was significantly different from zero (*r* = 0.41, Wilcoxon signed-rank test). **i** Z-scored firing rates of mouse-A cells and neither cells during social trials (Trial 1 + 2; left) and the control trial (Trial 3; right). Trial 1+2: Unit Type × Area, *F*_(1,69)_ = 96.6, *P* = 9.5 × 10^−15^, *η*^2^ = 0.35, partial *η*^2^ = 0.58; ***P = 3.3 × 10^−9^, *r* = 0.70 (A), 9.3 × 10^−8^, *r* = 0.63 (B). Mouse-A: ***P = 9.6 × 10^−10^, *r* = 0.73 (A vs B), 2.1 × 10^−7^, *r* = 0.62 (Center vs B); Neither: **P = 0.0081, *r* = 0.31 (A vs Center), *P = 0.024, *r* = 0.24 (Center vs B). Trial 3: UnitType × Area, *F*_(1,69)_ = 0.42, *P* = 0.52, *η*^2^ = 0.0040, partial *η*^2^ = 0.0061. All statistical tests are two-way repeated-measures ANOVA and Tukey–Kramer multiple comparison tests. **j** Firing timings of the two units shown in Fig. 1f, and relative social distance of the subject mouse. Shaded periods indicate that the subject was in the social zone around mouse-A (blue) or mouse-B (red). Ticks and filled circles represent spike timings of Unit #1 (blue) and #7 (red). **k** Correlations between social preferences and directional social preferences of pyramidal neurons (*n* = 75 cells). Whole arena, *r* = 0.65, *P* = 3.9 × 10^−10^; center zone, *r* = 0.35, *P* = 0.0021. **l** Comparison of directional social preferences. Comparison between neither vs mouse-A cells: **P =0.0022, *r* = 0.36, Wilcoxon rank sum test. Comparison between each group vs zero: mouse-A cells in whole arena, ***P = 3.7 × 10^−4^, *r* = 0.51 and mouse-A cells in center zone, *P = 0.026, *r* = 0.46, Wilcoxon signed-rank test. ns, not significant. **m** Preferred directions and mean resultant vectors of neither cells and mouse-A cells. The black arrow direction indicates mean direction preference of all neither cells and mouse-A cells. Arrow lengths of mean resultant vectors indicate the direction concentration parameter, kappa. Neither: *n* = 48 cells, mean direction = 317.0°, *P* = 0.31, V test for circular uniformity against 0°; mouse-A: *n* = 23 cells, mean direction = 10.6°, ***P = 1.3 × 10^−6^, V test for circular uniformity against 0°.

Mice display a natural inclination to approach a novel mouse rather than a familiar mouse [34]. The subject and a stimulator mouse were left in the home cage for 2 h of familiarization, which has been shown to be a sufficient amount of time for the emergence of both memory-dependent social discriminatory behavior and social memory neurons in the vCA1 [5]. After familiarization, we performed a social discrimination test (SDT) to assess the subject responses to the familiar mouse-A and a novel mouse-B (Fig. 1a). The SDT consisted of two consecutive 5 min trials in which the subject mouse was placed with mouse-A and mouse-B in counter-balanced positions (“social trials”) in a social arena (Supplementary Fig. 1a), followed by a 5 min “control trial” with two empty social chambers. We confirmed that the subject mouse interacted with the novel mouse-B for a significantly longer period of time than with the familiar mouse-A both by summed interaction time across social trials (mouse-A: 124.1 ± 7.6 s, mouse-B: 176.6 ± 13.8 s; *P* = 0.0054; Fig. 1c; *n* = 8 mice) and discrimination score (*P* = 0.0078; Fig. 1d), consistent with the findings of our previous study [5]. To analyze social memory representation at a fine temporal resolution, we implanted high-density, 64-site, four-shank silicon probes in the vCA1 (Fig. 1e) and recorded neural activity during the SDT (“online” recording) as well as during the subsequent 2 h resting period (“offline” recording). For each cell, we calculated a discrimination score based on the body position of the subject mouse during the SDT; cells that exhibited significant activation in response to social interactions either with the familiar mouse-A (e.g., Unit #1 in Fig. 1f, g) or the novel mouse-B (e.g., Unit #7 in Fig. 1f, g) were termed “mouse-A cells” and “mouse-B cells”, respectively. Additionally, each cell was classified either as an excitatory pyramidal neuron or an inhibitory interneuron based on its firing rate and trough-to-peak length of spike shapes (Supplementary Fig. 1c). Of the 104 units recorded over 11 sessions, 75 units were classified as putative excitatory pyramidal neurons, whereas 29 units were classified as putative inhibitory interneurons. Among the putative pyramidal neurons, we identified 31.1% as mouse-A cells and 4.0% as mouse-B cells, and found that their population activity indicated a significant preference for the familiar mouse-A (*P* = 4.9 × 10^−4^; Fig. 1h), similar to our previous findings based on Ca^2+^ imaging [5]. In contrast, the familiar mouse-A and novel mouse-B cell fractions among the putative inhibitory interneurons did not significantly differ (mouse-A cells 14.3%; mouse-B cells 7.1%; *P* = 0.66; Supplementary Fig. 1d).

To confirm that the observed neuronal activity was specific to social memory, we implemented a novel object recognition (NOR) test with the same time-course of the SDT (*n* = 3 sessions from 2 mice; Supplementary Fig. 2a). We found that, although a small number of neurons exhibited significant preferences for the familiar or a novel object (Supplementary Fig. 2b), the overall fraction of the stimulus-selective neurons was significantly smaller in NOR than in SDT (*P* = 5.1 × 10^−10^; Supplementary Fig. 2c).

### Firing properties of vCA1 neurons during online social experiences

We then carefully examined the in vivo firing properties of vCA1 neurons during the SDT. Cells classified neither as mouse-A cells nor as mouse-B cells (“neither cells”) were equally active when the subject mouse approached either mouse-A or mouse-B. In contrast, mouse-A cells were more active when the subject mouse approached mouse-A and, conversely, were less active when subject mouse approached mouse-B (Fig. 1i and Supplementary Fig. 1). When the subject mouse explored the arena in the absence of any social targets (Trial 3), the activity of the mouse-A cells was similar to that of the neither cells. These data suggest that a fraction of vCA1 cells is adaptively modulated to be activated or inactivated by the presence of social cues, which gives rise to the specific responses to a memorized social target.

Closer investigation revealed that social cells fired both when the subject mouse was interacting with the preferred social target and when approaching it (Fig. 1j), which led us to examine the neuronal activity patterns in terms of the relative direction of the subject towards either social target in the arena. By calculating the directional social preference score of each cell, for which firing was considered only when the subject was oriented towards the social targets, we observed a significant positive correlation between the social preference scores and the directional social preference scores (Fig. 1k, left). This correlation was maintained even when we narrowed down the criteria to include activities recorded when subjects were in the center zone of the arena (Fig. 1k, right), indicating that vCA1 neuronal activity related to social experiences appeared to be modulated not only by close interaction but also by distant recognition of social targets. Indeed, familiar mouse-A cells, determined based on discrimination scores, showed a significant directional preference as a population toward mouse-A throughout the arena (−0.21 ± 0.05, *P* = 0.0024) as well as in the center zone (−0.18 ± 0.07, *P* = 0.026; Fig. 1l). Directional preferences relative to the social target also confirmed that the mouse-A cells preferentially fired when the subject was oriented toward mouse-A (Fig. 1m).

### Firing patterns of vCA1 neurons during offline SPW-R events

**Fig. 2:**
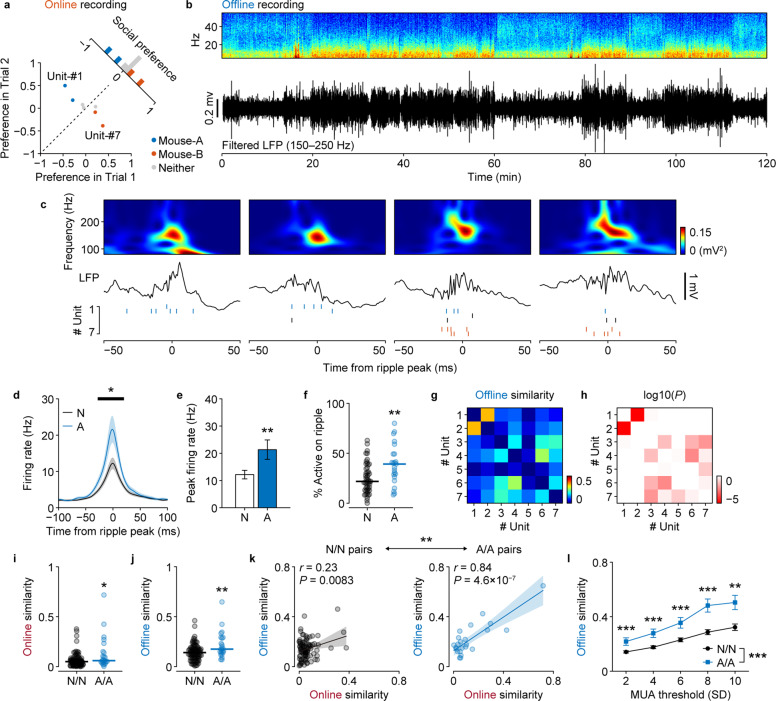
Neurons with similar social representations are co-activated during offline SPW-Rs. **a** Social preferences of example neurons recorded in the same session. **b** Example power spectrum and filtered ripple band trace of the LFP recorded in the home cage. **c** Representative SPW-Rs and raster plots of the units recorded during the same session as **a**. Black, blue, and red ticks indicate spikes of neither, mouse-A, and mouse-B neurons, respectively. **d** Mouse-A neurons (blue) had significantly elevated peak firing rates during SPW-Rs compared with the neither neurons (black). The horizontal red line indicates the time period where the firing rate was significantly different (*P* < 0.01, permutation test). The shaded areas signify the SEMs. **e** Comparison of firing rates at ripple peaks. neither cells (N): *n* = 48 cells, 12.2 ± 1.6 Hz; mouse-A cells (A): *n* = 23 cells, 21.3 ± 3.6 Hz; **P = 0.0037, Cohen’s *d* = 0.72, Student’s *t* test. **f** The fraction of SPW-Rs for which a cell fired at least one spike, averaged across all cells. **P = 0.0048, *r* = 0.33, Wilcoxon rank-sum test. Pairwise similarities of unit activities during SPW-Rs (**g**) and their statistical significance (**h**; permutation test). Note that units are sorted according to the social preferences plotted in **a**. Comparison of the online (**i**) and offline (**j**) activity similarity between N/N (*n* = 92) and A/A (*n* = 23) cell pairs. **P = 0.017, *r* = 0.22 and ***P = 0.0045, *r* = 0.26, Wilcoxon rank-sum test. **k** Correlations between online and offline activity similarity. **P = 0.0058, permutation test (Supplementary Fig. 4). **l** Quantitative measurement and comparison of the offline similarities at different threshold MUA factors between N/N (*n* = 92) and A/A (*n* = 23) cell pairs. Main effect of Group, *F*_(1,103)_ = 19.4, *P* = 2.6 × 10^−5^, *η*^2^ = 0.022, partial *η*^2^ = 0.16, Group × Threshold, *F*_(1,103)_ = 17.0, *P* = 7.6 × 10^−5^, *η*^2^ = 0.021, partial *η*^2^ = 0.14, two-way repeated measures ANOVA. ***P < 0.001, *r* = 0.25–0.41, Tukey–Kramer multiple comparison test.

To determine how the activities of vCA1 social memory neurons (Fig. 2a) are coordinated and organized during SPW-R events, we investigated the firing patterns of pyramidal cells during offline recordings (Fig. 2b). In addition to the expected increase in delta power (1–4 Hz), which indicated that the subject was resting, a concurrent increase in ripple power (150–250 Hz) was observed (Fig. 2b and Supplementary Fig. 3). Across all sessions, we consistently observed that SPW-R events were accompanied by multi-unit activities during the offline recordings (Fig. 2c; 1.1 ± 0.5 × 10^3^ events per 2 h, *n* = 11 sessions). The familiar mouse-A cells showed a significantly higher firing rate around ripple peaks (Fig. 2d,e) and participated in a larger fraction of ripples (Fig. 2f) compared to neither cells, indicating that cells encoding social representations are preferentially reactivated following social experiences.

To measure the synchrony between cells during offline SPW-R events, we calculated the cosine similarity between pairs of spike count vectors. When the recorded units were sorted according to the discrimination scores (Fig. 2g), the rendered square matrix of the similarity values clearly showed that a pair of familiar mouse-A cells (#1 and #2) were highly synchronized, to the exclusion of coactivation with other cells (Fig. 2g). In contrast, a pair of novel mouse-B cells (#6 and #7) were activated together with other cells during SPW-R events above the probability threshold calculated based on random shuffling (Fig. 2h), suggesting that the preferential reactivation of social memory neurons takes place at the population level. Likewise, by calculating the cosine similarity between spike count vector pairs in terms of video frames (25 Hz) during the online recordings, we found that pairs of mouse-A cells showed higher similarities than pairs of neither cells during both online (Fig. 2i) and offline recording (Fig. 2j). While pairs of both neither cells and familiar mouse-A cells showed significant positive correlations between online and offline similarities, the Pearson’s correlation coefficient of mouse-A cells was significantly higher than that of neither cells (*P* = 0.0058; Fig. 2k and Supplementary Fig. 4). Upon varying the threshold of the multi-unit activity (MUA) during SPW-Rs, we found that SPW-Rs with higher neuronal activities contained more correlated information content, and that this relationship was more prominent among mouse-A cells (*n* = 23 pairs) than neither cells (*n* = 92 pairs) (Group, *P* = 2.6 × 10^−5^; Group × Threshold, *P* = 7.6 × 10^−5^; two-way repeated measures ANOVA; Fig. 2l), again indicating that social memory was consolidated by the prioritized replay of specific neuronal ensembles.

### Firing sequence of social memory neurons

**Fig. 3:**
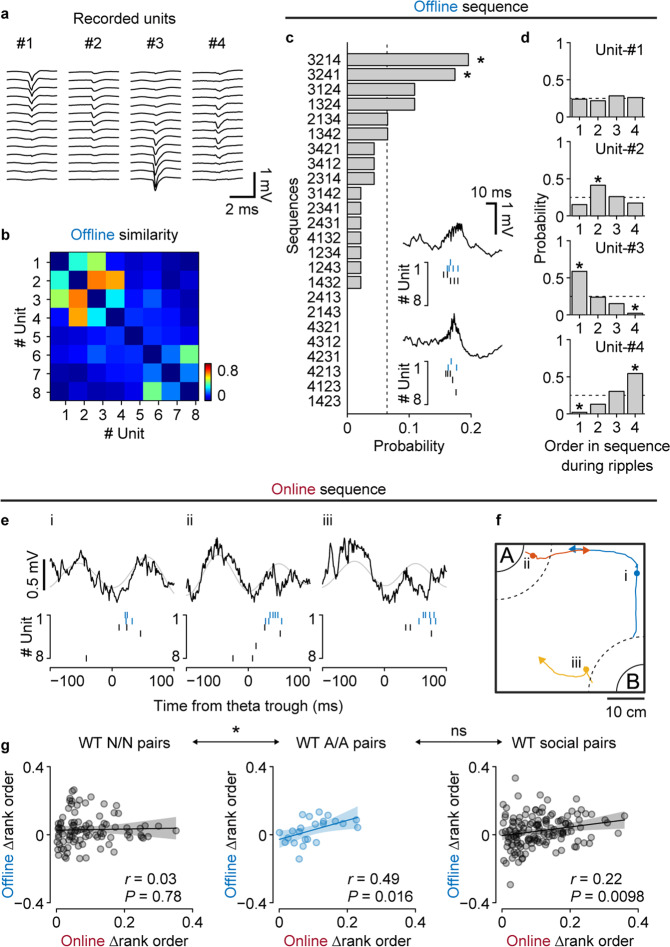
Spike sequences during online theta cycles and offline SPW-Rs. **a** Example four units recorded in the same session. **b** Activity similarity during offline SPW-Rs. Unit numbers correspond to those in **a**, and the units are sorted according to the social preferences. **c** Occurrence probability of the (4! = 24) possible neuronal sequences. The dotted line indicates chance level. **d** Probabilities that each unit was observed on a specific order within sequences. Dotted lines and gray bands indicate the chance levels. The asterisks in **c** and **d** represent motifs that are significantly more abundant or sparse than the chance (*p* < 0.05; determined by two-sided binomial test). **e** Example theta events during the SDT and simultaneously recorded spike sequences. Black and gray curves represent wide-band LFPs and theta (6‒12 Hz) band signals, respectively. **f** Behavioral trajectories around the theta events shown in **e**. Circles depict the positions where the events occurred, and lines show behavioral paths of the subject 1.5 s before and after the events. Note that all example theta events occurred while either interacting with (**b**) or approaching (**a** and **c**) the familiar social target, mouse-A. **g** Correlations between the differences of mean rank orders during online theta cycles and offline ripples. Orders within each cell pair were determined so that the Δonline mean rank orders always had a positive value. Pairs including at least one mouse-A or mouse-B cell were considered social pairs. Pairs of neither cells (N/N): *n* = 92 pairs, pairs of mouse-A cells (A/A): *n* = 23 pairs, social: *n* = 140 pairs.

To examine whether social memory neurons were analogously organized sequentially, we analyzed the temporal structure of vCA1 firing activities during offline SPW-R events. As a representative example, we recorded eight putative pyramidal cells and identified two of them as familiar mouse-A cells (Unit-#1 and 2; Fig. 3a). When their offline similarities were sorted according to social preference scores, the square matrix showed that four cells, including the mouse-A cells, were synchronously activated during SPW-R events (Fig. 3b). By examining the temporal orders of the first spike of each unit observed during SPW-Rs, we observed that two sequences out of the 24 possible variations had a significantly higher probability of occurrence than that of a random event (Fig. 3c) and that the members of the spike sequence had respective preferred orders (Fig. 3d).

To determine whether the spike sequences detected during offline SPW-Rs reflected the temporal structure of neuronal activities during online social experiences, we investigated the firing patterns of vCA1 neurons in terms of individual theta (6‒12 Hz) cycles during the SDT, and found that similar activity patterns were preserved (Fig. 3e). Interestingly, these theta-locked sequences arose while the subject was approaching (Fig. 3e, i and iii) or interacting with (Fig. 3e, ii) the familiar social target, consistent with our observation that social memory neurons could respond to preferred social targets that are at a distance (Fig. 1). To systematically compare the relationship between sequential ordering of neurons during individual online theta cycles and offline SPW-R events across data sets, we calculated the mean rank order during cycles/events [15] and the differences between each unit pair. We found that pairs of mouse-A cells, but not pairs of neither cells, showed a significant positive correlation between online and offline sequential ordering (Fig. 3g). The positive correlation still existed when pairs consisting of at least one social cell (namely one mouse-A or mouse-B cell) were incorporated. These results suggest that social representations in the vCA1 are temporally orchestrated at the population level centering around the social memory neurons, which maintain the sequential consistency from online social experiences to the offline resting period.

### Impaired social memory and disrupted spike sequences in *Shank3*-KO mice

**Fig. 4:**
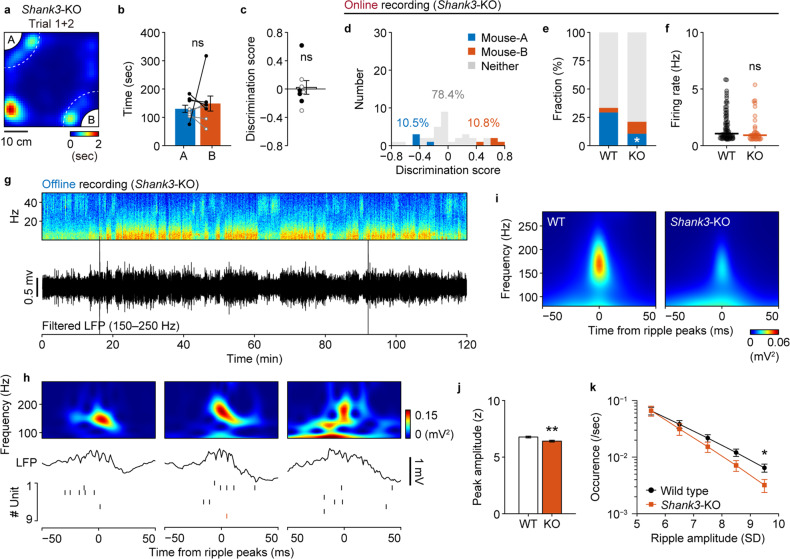
Impaired social memory and altered vCA1 activity of *Shank3*-KO mice. **a** Representative heat map of occupancy time during Trial 1 and Trial 2 with a *Shank3*-KO mouse. Note that the result of Trial 2 is rotated and superposed onto that of Trial 1. Comparison of duration spent in the social zone (**b**) and the discrimination score (**c**). *n* = 8 mice. *P* = 0.60, Cohen’s *d* = 0.19, paired *t*-test (**b**) and *P* = 0.74, *r* = 0.12, Wilcoxon signed-rank test (**c**). **d** Discimination scores of recorded pyramidal neurons (*n* = 37 cells). Blue, mouse-A cell (*n* = 4 cells, 10.8%); red, mouse-B cell (*n* = 4 cells, 10.8%); gray neither neuron (*n* = 29 cells, 78.4%). **e** Comparison of the fractions of neurons with significant social representation between WT and *Shank3*-KO mice. *P = 0.019, Z test of proportions. **f** Comparison of firing rates (*P* = 0.09, Wilcoxon rank-sum test). **g** Example power spectrum and filtered ripple band trace of the LFP recorded in the home cage. **h** Representative SPW-Rs and raster plots of the units recorded during the same session as in **g**. Black and red ticks indicate spikes of neither and mouse-B neurons, respectively. **i** Representative SPW-R triggered spectrograms averaged across entire representative sessions from WT and KO mice. **j** Averaged peak ripple amplitude was significantly lower in KO mice (*n* = 7 sessions) mice than in WT mice (*n* = 11 sessions). **P = 0.0056, *t*_(16)_ = 3.17, Cohen’s *d* = 1.63, Student’s *t* test. **k** *Shank3*-KO mice generated fewer ripples with large amplitudes. *P < 0.05, *r* = 0.47, Tukey–Kramer multiple comparison test.

To validate the reported impairment of social novelty preference in ASD model *Shank3*-KO mice [23], we conducted our SDT as described in Fig. 1a. As expected, the duration of approach of *Shank3*-KO mice for both the familiar mouse-A and the novel mouse-B was comparable (Fig. 4a, b), with a discrimination score not significantly different from zero (*P* = 0.74; Fig. 4c). Consistent with these behavioral abnormalities, the proportion of identified mouse-A cells in *Shank3*-KO mice was significantly smaller than that in wild-type mice (*Shank3*-KO: mouse-A cells 10.8%, mouse-B cells 10.8%, neither cells 78.4%; *P* = 0.019; Figs. 1h, 4d, e), although the firing rate of recorded pyramidal cells did not differ between wild-type and *Shank3*-KO mice (Fig. 4f). We also observed that SPW-Rs in *Shank3*-KO mice during offline recording (Fig. 4g) resembled those observed in wild-type mice (Fig. 4h) in terms of the ripple rate (WT: 0.15 ± 0.07 Hz, *n* = 11 sessions; KO: 0.13 ± 0.07 Hz, *n* = 7 sessions; *P* = 0.33), duration (WT: 53.5 ± 7.0 ms, KO: 54.0 ± 5.1 ms; *P* = 0.89), and peak frequency (WT: 155 ± 2.3 Hz, KO: 154 ± 1.7 Hz; *P* = 0.80) (Supplementary Fig. 5c). Proportion of time spent resting (WT: 64 ± 3.4%, KO: 75 ± 7.1%; *P* = 0.14) and the power spectrum density of the LFP signals during the offline recordings were also comparable (Supplementary Fig. 5b). However, we found that the *z*-scored peak amplitude of SPW-R events was significantly lower in *Shank3*-KO mice compared to that in wild-type mice (WT: 6.8 ± 0.08, KO: 6.4 ± 0.09; *P* = 0.0056; Fig. 4i, j). The difference was ascribed to the decrease in larger SPW-R events (Fig. 4k), which has been shown to be related to learning and memory consolidation [35].

**Fig. 5:**
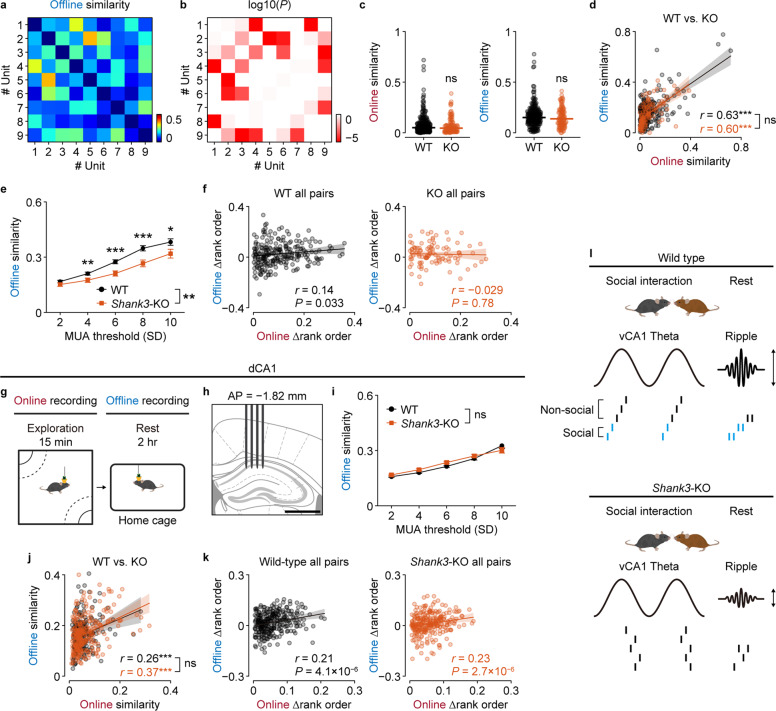
Disrupted spike sequences and ripple phase locking in *Shank3*-KO mice. Pairwise similarities of unit activities during SPW-Rs (**a**) and their statistical significance (**b**; based on a permutation test). Units are sorted according to the social preferences. **c** Comparable online (left) and offline (right) activity similarity between pairs from WT (*n* = 237 pairs, 11 sessions) and KO (*n* = 100 pairs, 7 sessions) mice. **d** Correlations between online and offline activity similarity of simultaneously recorded cell pairs from WT (*n* = 206 pairs) and KO (*n* = 100 pairs) mice. **e** Quantitative measurement and comparison of offline similarities at different MUA threshold factors between WT and KO mice. Main effect of Genotype, *F*_(1,304)_ = 9.7, *P* = 0.0020, *η*^2^ = 0.0043, partial *η*^2^ = 0.029; Genotype×Threshold, *F*_(1,304)_ = 9.8, *P* = 0.0019, *η*^2^ = 0.0047, partial *η*^2^ = 0.030. ***P < 0.001, **P < 0.01, and *P < 0.05, *r* = 0.12–0.20, Tukey-Kramer multiple comparison test. **f** Correlations between the differences of mean rank orders during online theta cycles and offline ripples. **g** Schematic of the behavioral paradigm. **h** A four-shank silicon probe was implanted in the dorsal CA1 of freely moving mice. **i** Quantitative measurement and comparison of offline similarities at different MUA threshold factors between WT (*n* = 2 mice, *n* = 338 pairs, 4 sessions) and KO (*n* = 2 mice, *n* = 366 pairs, 4 sessions) mice. Main effect of Genotype, *F*_(1,702)_ = 0.694, *p* = 0.41, *η*^2^ = 9.4 × 10^−5^, partial *η*^2^ = 9.4 × 10^−4^; Threshold, *F*_(1,702)_ = 2244.2, *P* = 7.8×10^−221^, *η*^2^ = 0.39, partial *η*^2^ = 0.76; Genotype × Threshold, *F*_(1,702)_ = 0.296, *P* = 0.59, *η*^2^ = 5.2 × 10^−5^, partial *η*^2^ = 1.0 × 10^−4^. **j** Correlations between online and offline activity similarity of simultaneously recorded cell pairs from WT (*P* = 1.7 × 10^−4^; *n* = 210 cells) and KO (*P* = 7.2 × 10^−10^; *n* = 265 cells) mice. ns, *P* = 0.19, permutation test. **k** Correlations between the differences of mean rank orders during online theta cycles and offline ripples. ns, *P* = 0.86, permutation test. **l** Schematic representation of the findings.

Finally, we investigated the offline neuronal activity in the vCA1 of *Shank3*-KO mice and examined its consistency with the activity patterns during online social experiences (Fig. 5a, b). Contrary to our expectations, both online and offline similarities were indistinguishable from those of wild-type mice (Fig. 5c) and exhibited a significant positive correlation, comparable to that in the wild-type mice (Fig. 5d). However, by varying the threshold, we found that SPW-Rs with higher MUAs did not give rise to more correlated neuronal activities in *Shank3*-KO mice compared to that in wild-type mice (Group, *P* = 0.0020; Genotype×Threshold, *P* = 0.0019; two-way repeated-measures ANOVA; Fig. 5e). Furthermore, the correlation between online and offline sequential ordering was disrupted in *Shank3*-KO mice (Fig. 5f). Contrary to the activity similarity (Figs. 2l and 5e), the spike sequence consistency remained stable upon varying the threshold of MUAs during SPW-Rs (Supplementary Fig. 6c–f). These results suggest that both rate and temporal coding consistency of neuronal activities between online social experiences and offline resting period are impaired in *Shank3*-KO mice.

To examine whether these physiological alterations observed in *Shank3*-KO mice are specific to the vCA1, we recorded neural activity in the dorsal CA1 (dCA1) during spatial exploration and resting period after the exploration (Fig. 5g–k, Supplementary Fig. 5). We observed SPW-Rs with comparable number of events (WT: 0.38 ± 0.03 events/s, KO: 0.36 ± 0.04 events/s; *P* = 0.66), duration (WT: 60.0 ± 1.8 ms, KO: 60.6 ± 0.6 ms; *P* = 0.74), and peak frequency (WT: 144.0 ± 0.7 ms, KO: 144.6 ± 1.2 ms; *P* = 0.69) between wild-type mice and *Shank3*-KO mice, although the KO spent significantly longer slow-wave sleep (SWS) period (WT: 64.9 ± 4.1%, KO: 79.0 ± 3.3%; *P* = 0.037) (Supplementary Fig. 5d). Comparable activity similarity both during online and offline recordings (Supplementary Fig. 5h) and their significant positive correlations (*P* = 0.19; Fig. 5j) were also observed. Contrary to the vCA1, SPW-Rs accompanying higher MUAs in the dCA1 gave rise to more correlated neuronal activity both in the wild-type mice and *Shank3*-KO mice (Fig. 5i). Furthermore, we observed that the significant positive correlation of mean rank order differences between online theta cycles and offline ripples was preserved in *Shank3*-KO mice (*P* = 0.86; Fig. 5k). These results suggest that the disruption of spike sequence consistency observed in the vCA1 of *Shank3*-KO mice is not a general phenomenon across the dorsoventral axis of the hippocampus, but it is specific to its ventral part.

Temporally precise, phase-locked inhibition supports hippocampal ripple generation [36, 37]. To investigate the neural substrate of disrupted sequential ordering, we compared the phase-locking of spike timings to ripple oscillations across groups (Supplementary Fig. 7). While the ripple oscillation induced a significant (*P* < 0.001) phase-locking in both the majority of pyramidal cells (WT: 156.3 ± 2.7°, 86.5% significantly phase-locked, *n* = 74 cells; KO: 149.6 ± 2.1°, 97.3% significantly phase-locked, *n* = 37 cells; *P* = 0.08, Z test of proportions) and interneurons (WT: mean 176.7 ± 4.3°, 75.0% significantly phase-locked, *n* = 28 cells; KO: mean 181.7 ± 8.6°, 80.0% significantly phase-locked, *n* = 20 cells; *P* = 0.68, Z test of proportions) across both genotypes, the strength of phase-locking (expressed as mean resultant length) was specifically decreased among interneurons (WT: 0.32 ± 0.03, *n* = 21 cells; KO: 0.21 ± 0.02, *n* = 16 cells; *P* = 0.0017) but not pyramidal cells (WT: 0.38 ± 0.03, *n* = 64 cells; KO: 0.39 ± 0.02, *n* = 36 cells; *P* = 0.84) in *Shank3*-KO mice. These results indicate that a loss of phase-locked inhibitory inputs onto pyramidal neurons may lead to an impairment of precise spike timings and their temporal sequences in social cell assemblies.

## Discussion

A growing body of work examining social memory functions has focused primarily on two pivotal sub-regions of the hippocampus, the vCA1 and dorsal CA2 (dCA2) [3, 38, 39]. A recent study reported that dCA2 neurons respond to novel conspecifics during social interaction and are reactivated during SPW-Rs [40]. In contrast, our study revealed that the instantaneous emergence of neuronal activities associated with a novel individual during social exploration was not detected in vCA1 neurons, whereas robust online activation and later offline reactivation of neuronal ensembles were observed in response to familiarized individuals (Figs. 1h and 2). When encoding spatial representations, place-cell activity in the hippocampus stabilizes during spatial exploration [41], and recorded neurons in the dCA1 require a certain minimum level of experience to establish stable spatial representations [42]. Analogously, to form and store social memories, neural ensembles in the vCA1, but not those in the dCA2, may require social interaction of sufficient duration to develop stable and observable neuronal activities that represent familiar individuals. Since CA2 neuronal projections innervate downstream hippocampal structures along the septotemporal axis [43], social information processed in the dCA2 appears to contribute to the consolidation of social memories in the vCA1 by propagating SPW-Rs [17]. As synapses on CA2 pyramidal neurons in adult mice are more resistant to the induction of long-term potentiation (LTP) compared to those on CA1 neurons [44], dCA2 and vCA1 neurons may cooperate while executing distinct functions, such as processing of social information in the dCA2 and encoding and storing of social memories in the vCA1.

A recent study using Wistar rats reported that more than 50% of pyramidal cells in the vCA1 were strongly activated by the presence of familiar conspecifics [6], which comprise a considerably larger population than the one we observed. Our criteria for identifying social memory neurons, which employ the relative difference of neuronal activity around the social stimuli, did not aim to capture social neurons that respond to any conspecifics regardless of whether they are familiar or not. Indeed, some neurons which were classified as non-social according to our criteria were activated around both social stimuli (Supplementary Fig. 1h). Moreover, social memory neurons identified by our criteria (i.e., mouse-A cells) showed not only selective excitation around the familiar mouse, but also inhibition around the novel mouse (Fig. 1i). This is probably due to the activation of parvalbumin interneurons [45], which may contribute to the fine-tuning of social memory neurons.

Memory-guided discriminatory social behavior is supported by projections from vCA1 social engram neurons to the nucleus accumbens (NAc) [5]. A recent study demonstrated that SPW-Rs occur asynchronously in the dorsal and ventral hippocampus during the awake state and modulate distinct populations of NAc medium spiny neurons (MSNs) [46]. It has also been reported that dorsal but not ventral hippocampal SPW-Rs activate NAc neurons that encode information related to spatial and reward-related information [46]; therefore, ventral hippocampal SPW-Rs may specifically convey social information to MSNs in the NAc to establish a neural circuit that supports memory-guided social behavior.

Sequential activation of neuronal ensembles appears to serve as a general mechanism for the consolidation of semantic memories in the hippocampus [47]. Analogous to the place cell sequences in the dCA1, the sequential firing of vCA1 social memory neurons could reflect a certain type of information structure. One possible hypothesis is that the firing sequence might be organized according to the perceptual origin of social stimuli. Both in rodents and humans [48], social recognition and its memorization are supported by multimodal sensory inputs such as visual [49] and olfactory [50] stimuli. Indeed, “concept cells” in the human hippocampus that encode social memory can fire selectively not only in response to pictures of a specific person but also in response to other sensory modality cues such as the pronounced or written name of that person [51]. The presence of social neurons that are preferentially activated in response to specific social targets, even when the test subjects were distant from the social interaction zone (Fig. 1j), indicates that some vCA1 social memory neurons are more specifically tuned to visual or auditory cues, which can be processed across distances, rather than olfactory cues, which require close proximity. Although male mice rarely emit ultrasonic vocalizations directed to other males even during direct interactions [52], which makes it unlikely that vocal communications were involved in social recognition in our study (as opposed to rats [6]), it is still possible that mice can utilize auditory cues for recognizing conspecifics, especially those who are of different sex. Tactile perception derived from direct interactions, such as facial touch, which has been shown to modulate neuronal activity in the vCA1 [6], also possibly supports distinguishing other individuals. Nevertheless, future studies are necessary to examine whether and how social cues that are represented through a single sensory modality activate vCA1 social neurons, presumably through the dCA2-vCA1 pathway [43] or the conventional vCA3-vCA1 pathway, in which pattern separation and completion are thought to occur [53].

In the dorsal hippocampus, forward replay is significantly more frequent than reverse replay [54, 55], which contributes to the spike sequence consistency between online theta cycles and offline ripples [56]. Similar differences in the occurrence rate would underlie the stable spike orders of social memory neurons (Fig. 5f). The absence of positive correlation between the spike sequence consistency and the ripple strength measured by MUAs may be attributed to the exponentially fewer SPW-Rs accompanying higher MUAs (Supplementary Fig. 6e), and to the decrease in the number of co-activated ripples on each cell pair (Supplementary Fig. 6f), which prevents robust readout of spike orders. The intact spike sequence consistency and the decreased ripple amplitude observed in the dCA1 (Fig. 5k and Supplementary Fig. 5f) indicate that the neural substrate of high-frequency oscillations and the temporal coding are different, or at least not identical. To consolidate an episodic memory, the offline reactivation of a neuronal ensemble must incorporate multiple features of the experience, including spatial, temporal, and social information. In line with this notion, our recordings captured sequential activities among both social and non-social neurons (Fig. 3). Neuronal recordings from a larger neuronal population could elucidate how the detailed contents of experiences are mapped onto sequential neural activities. Although we did not conduct recording experiments before the familiarization in this study, it will be interesting to investigate how social memory neurons emerge from preexisting network dynamics in the ventral hippocampus.

As in a human study that reported that adult patients with ASD present with deficiencies in their memory of faces and social scenes [25], we observed that homozygous *Shank3*-KO mice exhibited social memory impairments within our social discrimination paradigm (Fig. 4b, c). Consistent with this behavioral phenotype, a smaller proportion of vCA1 neurons in *Shank3*-KO mice responded to familiar individuals, even after forming social memory (Fig. 4d, e), although the overall firing rate of recorded pyramidal neurons was normal. How does the deficiency in Shank3 expression lead to these behavioral and physiological impairments? The induction of synaptic potentiation is thought to be a general mechanism underlying memory formation [57]. Several in vitro electrophysiological studies have reported that LTP is impaired in the hippocampal CA1 synapses of *Shank3*-KO mice, whereas long-term depression (LTD) remains unaffected [58]. Therefore, the synapses on social memory neurons in the vCA1 are likely to be similarly defective in terms of the induction of the necessary synaptic potentiation required to form a social memory.

More importantly, in our study, which focused on the neural coding that occurs during SPW-Rs, pairwise measures of unit coactivation revealed that *Shank3*-KO mice showed decreased coactivation of vCA1 neurons during SPW-Rs, likely due to the malformation of neural networks resulting from the impairment of LTP on vCA1 neurons. A series of studies have demonstrated that the appropriate balance between the LTP and LTD of CA1 neurons induced by SPW-Rs is crucial for memory formation and consolidation. The relative spike timings of dCA3 and dCA1 place cells during SPW-Rs induce synaptic potentiation on dCA1 neurons [59]. In contrast, SPW-Rs that occur during slow-wave sleep lead to spontaneous synaptic depression on dCA1 neurons, which is dependent on the N-methyl-D-aspartate receptor, whose inactivation results in impaired learning of new memories [14]. Schizophrenia model Calcineurin-KO mice, which are severely LTD-deficient at hippocampal synapses, showed impaired ripple-associated reactivation of spatial sequences [60] and deficits in hippocampus-dependent working and episodic-like memory tasks [61]. While genetic animal models of neurodevelopmental disorders do not fully recapitulate the complexity and the wide spectrum of behavioral phenotypes observed among people suffering from such diseases, our results using an ASD model mouse indicated that malformations of vCA1 neural ensembles are likely due to the disrupted temporal coding within SPW-Rs, which interferes with the formation of social memories and the elicitation of social discriminatory behaviors.

In conclusion, our study provides empirical evidence to suggest that a key concept of social memory representation involves neural ensembles in the vCA1 and that pathological involvement of the vCA1 may underlie the physiological characteristics of ASD.

## Supplementary information

SUPPLEMENTAL MATERIAL

## Data Availability

The data supporting this study are available from the corresponding author upon reasonable request.

## Code availability

The code for analyzing the data is available from the corresponding author upon reasonable request.
