# Supplementary material for: Disrupted social memory ensembles in the ventral hippocampus underlie social amnesia in autism-associated Shank3 mutant mice
Source: Mol Psychiatry. 2022 Feb 4;27(4):2095–105. doi: 10.1038/s41380-021-01430-5 (PMC9126818; doi:10.1038/s41380-021-01430-5)
Supplement: Supplementary file 1 — SUPPLEMENTAL MATERIAL [file 41380_2021_1430_MOESM1_ESM.pdf]

Materials and Methods

Surgical procedures

All surgical procedures were performed under 1–2% isoflurane anesthesia and with the mice mounted on a stereotaxic apparatus (SR-9M-HT; Narishige, Tokyo, Japan). Dental cement was used to attach a custom-made head frame onto a cleaned skull for the convenience of subsequent silicon probe implantation. Mice were individually housed after the surgery. After 1–2 weeks of recovery, mice were again mounted with the head frame, and 64-channel high-density silicon probes (A4x16-Poly2-5mm-20s-150-160; NeuroNexus, Ann Arbor, MI) targeting the right ventral hippocampus (AP −3.00–3.45 mm, ML +3.10 mm from bregma) or the left dorsal hippocampus (AP −1.82 mm, ML −1.00–1.45 mm from bregma) were implanted parallel to the midline. Probes were attached to movable microdrives and lowered gradually over the course of several days until the CA1 pyramidal layer was reached, determined by the appearance of hippocampal SPW-Rs and pyramidal cell activity. After every recording session, probes were further lowered by ~50 μm to avoid recording from the same neurons.

Social discrimination test (SDT)

Mice were individually habituated to the investigator by handling for several minutes on each of two consecutive days (Day-1 and Day-2). Habituation to the social arena and recording cable was performed for 5 min on each of two consecutive days (Day-2 and Day-3). The social arena was a white acrylic box (area, 38×38 cm^2, height, 30 cm); two custom-made social chambers with quadrant bottom shape (7.5-cm radius) printed with a 3D printer (Original Prusa i3 MK3S; Prusa Research Prague, Czechia) using white PETG filaments were placed at the opposite corners of the arena. A social chamber consists of

bars with ~1-cm gaps and a cap, which can confine a stimulus mouse inside while allowing animals to interact with each other through the gaps. Social chambers On Day-4, a stimulus mouse (C3H or BALB/c; A) was placed into the home cage of the subject mouse for familiarization for 2 hours. Five minutes later, the familiarized mouse (A) and a novel mouse (BALB/c or C3H; B) were placed in the left or right chamber, and the subject mouse was allowed to explore inside the arena for 5 min (A–B trial). This procedure was repeated by alternating the position of the two stimulus mice (B–A trial) and then in their absence (E–E trial). Trials were repeated with 5-min intertrial intervals. Trials were video recorded at 25 frames per s from the top view of the arena using an area scan camera (acA1300-60gmNIR; Basler, Ahrensburg, Germany) equipped with a Computer 4–8-mm C-Mount lens. Video recording system was controlled by Bonsai software 2.6.2 [62]. Immediately after the E–E trial (typically ~1 min), the subject mouse was moved back to the home cage and allowed to rest for 2 h. Five subjects repeated experimental sessions with at least a 24-h interval using a different group of stimulus mice. Novel object recognition (NOR) test was performed following the same time-course of the SDT but using inanimate objects. All objects were in different shapes, made of distinct materials (wood, glass, metal, or plastic), and about 10 cm tall and 6×6-cm footprint.

Histology

After completion of the experiments, mice were deeply anesthetized and transcardially perfused with phosphate-buffered saline and then 4% paraformaldehyde. Thereafter, brains were dissected out and post-fixed in 4% paraformaldehyde at 4 °C overnight, and fixed samples were sectioned into 100-μm coronal slices using a vibratome (Leica, VT1000S; Leica Microsystems, Wetzlar, Germany). For verifying the electrode tracks, images were acquired using a microscope (BZ-X710; Keyence, Tokyo, Japan).

Social activity of hippocampal units

Discrimination score for each unit was computed as $(f_A - f_B)/(f_A + f_B)$, where f_A and f_B were the firing rates of a unit when the subject was located in the social zone around the stimulator mouse A or B, respectively. Significance of the calculated score was determined by a permutation test of spike counts in each video frame (repeated 10,000 times). The *P* value represents the proportion of shuffled values larger than the actual observed value. Neurons with a significant ($P < 0.05$ by permutation test) discrimination score were classified either as mouse-A neuron (score < 0) or mouse-B neuron (score > 0), respectively. The egocentric directional tuning function for each unit was the ratio between the peaks of spike count and total time spent in each direction in bins of 5° and smoothed with a Gaussian kernel of 2 bins.

LFP spectrum analysis

Root mean square (RMS) of the LFP signal across channels having pyramidal cells was calculated at each time point. The signal was then binned into 10-s epochs and the instantaneous power spectrum density (PSD) was estimated using Welch's method with a Hamming window (size = 1.33 s, overlap = 0.67 s). The obtained PSD was used to display spectrograms across offline recordings, to compare the spectral structure of the LFP between genotypes, and to assess the behavioral state during offline recordings as described below. To display spectrograms of SPW-Rs, continuous wavelet transform (CWT) was applied to unfiltered LFP signals using complex Morlet wavelets with a parameter of 7. Peak ripple frequency was identified as the frequency bin higher than 100 Hz in which the CWT has the largest value on the ripple peak timings.

Sleep analysis

To assess the behavioral state during offline recordings, signals from a 3-axis accelerometer on the headstage were utilized to calculate the derivatives of roll and pitch, which reflect angular velocities of the subject's head. The L2 norm of these derivatives was used as a proxy for the overall movement of the subject. The signal was then binned into 10-s epochs and sleep epochs were defined as the period of sustained immobility (0.1 arbitrary unit/s). The delta (2–5 Hz) power calculated by Welch's method was referenced to confirm that the movement signal recapitulated the physiological sleeping state.

Ripple event detection

The LFP signal was bandpass filtered (150–250 Hz) on each channel having pyramidal neurons, and the root mean square across channels was calculated at each time point. The power was then Gaussian smoothed (4 ms standard deviation [SD]). Epochs with a peak power exceeding the mean by at least 5 SD for at least 15 ms were detected. The sample points at which the power reduced below 1 standard deviation were determined as the points of onset and offset of the epochs. Ripple pairs with peaks closer than 50 ms were merged into single events.

62. Lopes G, Bonacchi N, Frazão J, Neto JP, Atallah BV, Soares S, et al. Bonsai: an event-based framework for processing and controlling data streams. Front Neuroinform. 2015;9:7.

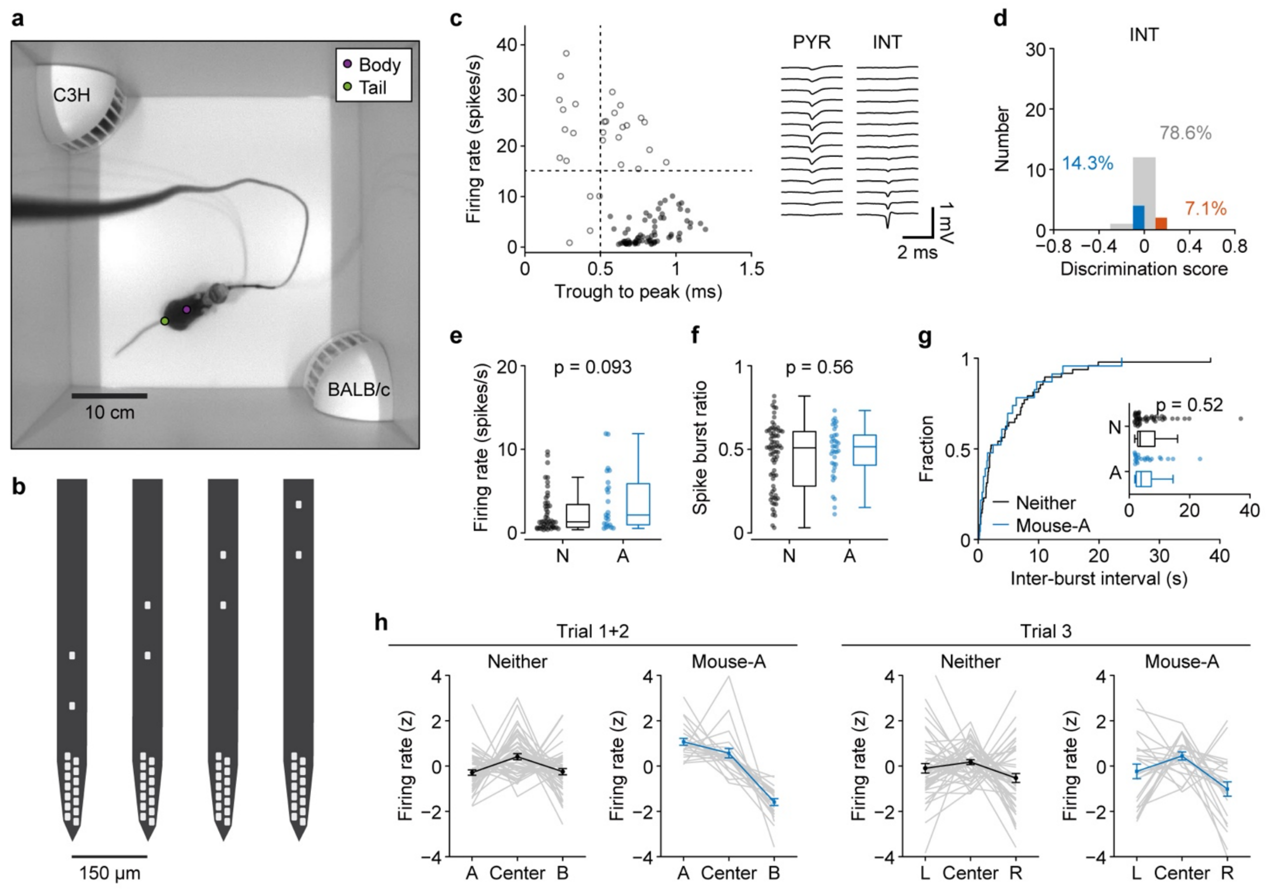

Supplementary Fig. 1 An apparatus of SDT and the properties of vCA1 social cells.

(**a**) A top view of the social arena with two social chambers during SDT. (**b**) An illustration of the probe tip. (**c**) Neurons with a low firing rate (< 15 Hz) and wide trough-to-peak spike shape length (> 0.5 ms) were classified as excitatory pyramidal neurons. (**d**) Discrimination scores of recorded interneurons (n = 29 cells). (**e**) Firing rate of neither cells (N; n = 48 cells) and mouse-A cells (A; n = 23 cells). (**f**) Proportion of spikes in bursts (spikes occurring with an inter-spike interval of 3–15 ms) of neither cells and mouse-A cells. (**g**) Distribution of inter-spike-intervals of neither cells and mouse-A cells. (**h**) Z-scored firing rates of mouse-A cells and neither cells during the social trials (Trial 1+2; left) and the control trial (Trial 3; right).

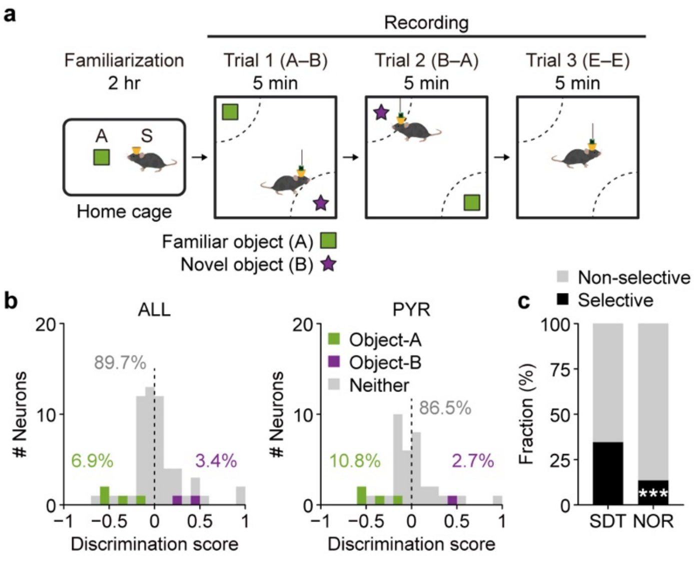

Supplementary Fig. 2 Novel object recognition (NOR) test.

(**a**) Schematic of the behavioral paradigm. After 2 h of familiarization between a subject mouse (S) and an object-A in a home cage, the subject mouse was allowed to explore a social arena where the familiar object-A and a novel object-B were placed. Dotted lines in the arena indicate the borders of interaction zones, which are identical to those used for SDT. (**b**) Discrimination scores of all recorded neurons (left; n = 57 cells) and putative pyramidal neurons (n = 37 cells). (**c**) Comparison of the fractions of neurons with significant social (obtained from the SDT) or object (obtained from the NOR) selective responses. ***$P = 5.1\times10^{-10}$, Z test of proportions.

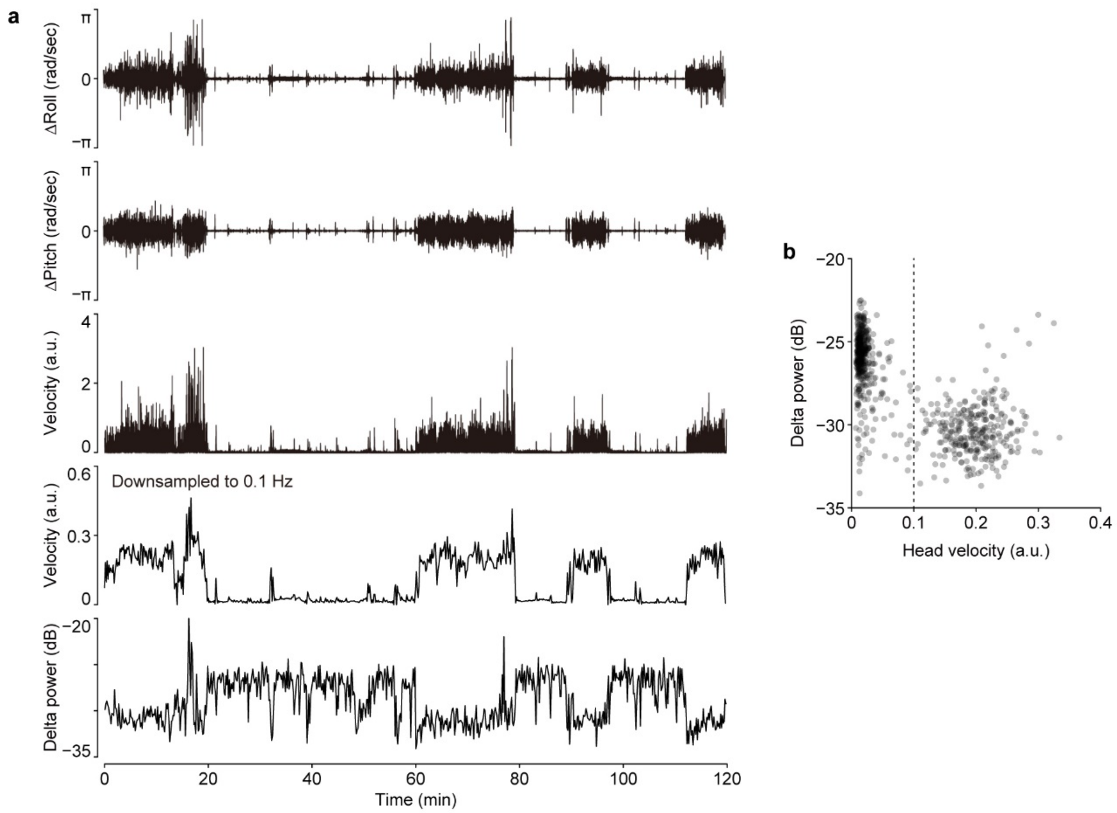

Supplementary Fig. 3 Sleep analysis and ripple event detection.

(**a**) Representative signals related to head movement. Signals from a 3-axis accelerometer mounted on the headstage were used to calculate the derivatives of roll and pitch of the subject's head, and the L2 norm of these derivatives was used as a proxy for the overall movement of the subject. (**b**) Relationship between head movement and the instantaneous delta power recorded from the vCA1. Bin length = 10 s. Epochs with head velocity less than 0.1 arbitrary unit (a.u.) were considered to be immobile, sleeping periods.

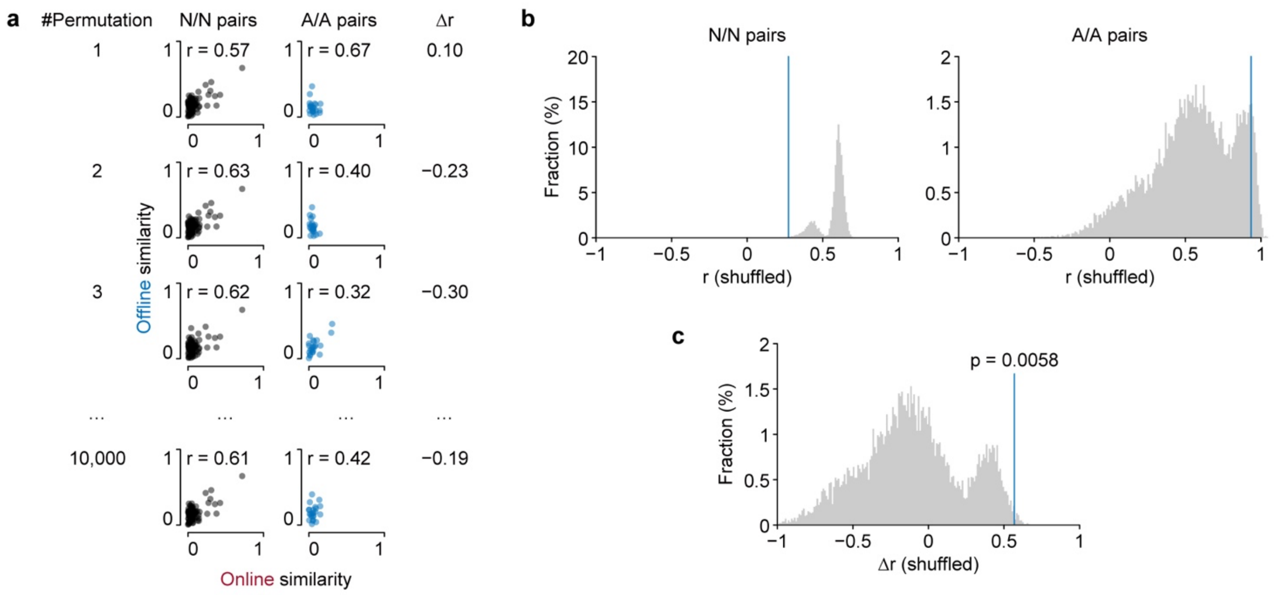

Supplementary Fig. 4 A permutation test for comparing correlation coefficients.

(**a**) Pairs of neither cells (n = 92 pairs) and mouse-A cells (n = 23 pairs) were shuffled and randomly divided into two groups with the same number of pairs with the actual data. Correlation coefficients for each shuffled group and the difference between them were calculated, and this procedure was repeated 10,000 times. (**b** and **c**) Frequency distributions of the coefficients calculated for the shuffled groups were compared with the original values to test two-tailed significance.

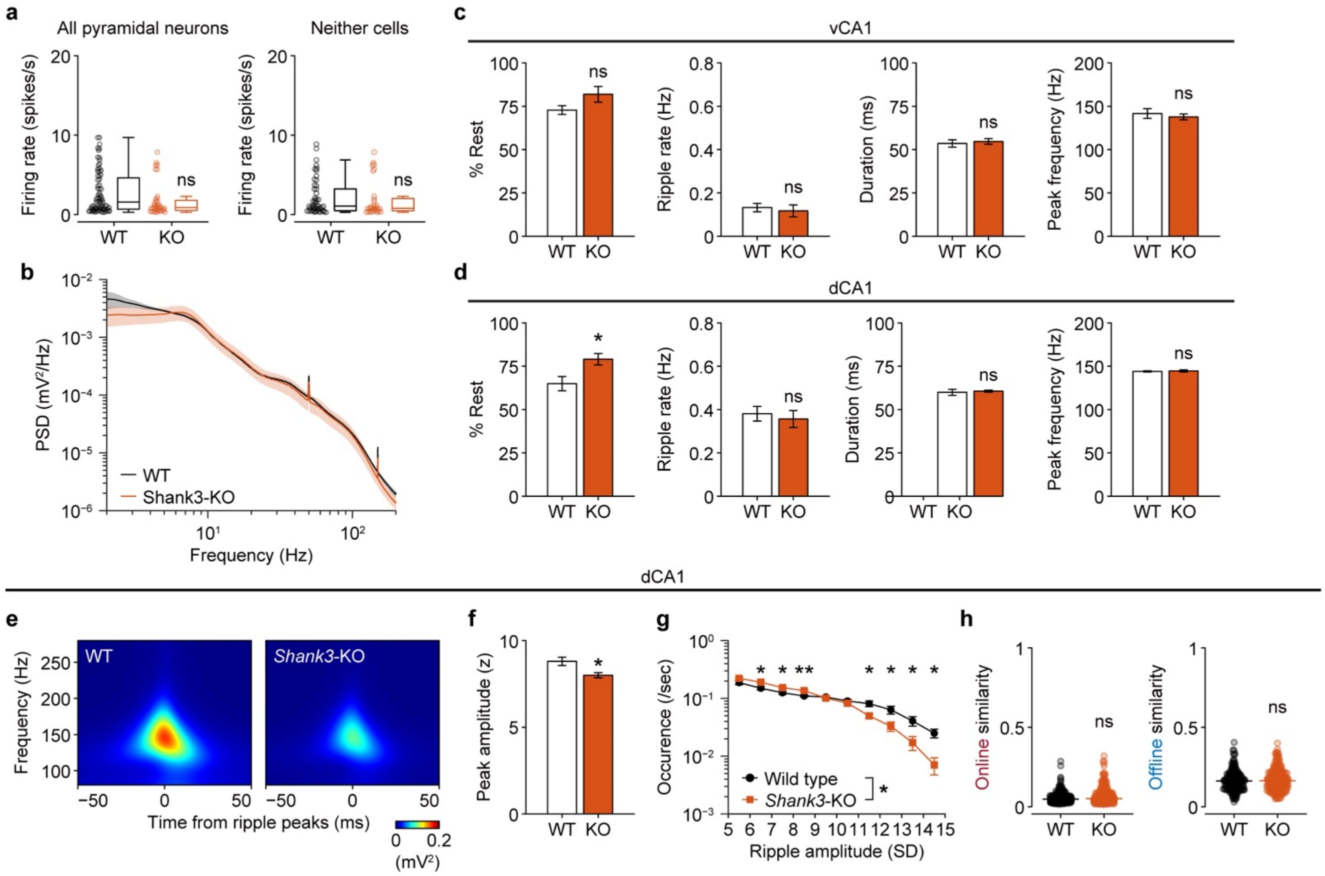

Supplementary Fig. 5 Electrophysiological properties of vCA1 neurons in wild-type and *Shank3*-KO mice.

(**a**) Comparison of the firing rate of all recorded pyramidal neurons (left) and neither cells (right) during the SDT between wild-type (n = 74 cells) and *Shank3*-KO (n = 37 cells) mice. ns, not significant by Wilcoxon rank-sum test. (**b**) Power spectrum density of the LFP signals recorded from the vCA1 of wild-type (n = 11 sessions) and *Shank3*-KO (n = 7 sessions) mice. (**c**) Proportion of time spent resting (WT: 63.9 ± 3.4 %, KO: 74.9 ± 7.1 %; P = 0.14, $t_{(6)}$ = −1.57, Cohen's d = 0.81, Student's t-test), ripple rate (WT: 0.15 ± 0.07 Hz, KO: 0.13 ± 0.07 Hz; P = 0.53, $t_{(16)}$ = 0.65, Cohen's d = 0.33, Student's t-test), ripple duration (WT: 53.5 ± 7.0 ms, KO: 54.0 ± 5.1 ms; P = 0.89, $t_{(16)}$ = −0.15, Cohen's d = 0.074, Student's t-test), and peak ripple frequency (WT: 141.6 ± 5.6 Hz, KO: 137.7 ± 3.3 Hz; P = 0.62, $t_{(16)}$ =

0.51, Cohen's *d* = 0.26, Student's *t*-test) were comparable between WT and KO mice. (**d**) Proportion of time spent resting (WT: 64.9 ± 4.1 %, KO: 79.0 ± 3.3 %; $P = 0.037$, $t_{(6)} = -2.68$, Cohen's $d = 2.18$, Student's *t*-test), ripple rate (WT: 0.38 ± 0.03 Hz, KO: 0.36 ± 0.04 Hz; $P = 0.66$, $t_{(6)} = 0.47$, Cohen's $d = 0.38$, Student's *t*-test), ripple duration (WT: 60.0 ± 1.8 ms, KO: 60.6 ± 0.6 ms; $P = 0.69$, $t_{(6)} = -0.35$, Cohen's $d = 0.29$, Student's *t*-test), and peak ripple frequency (WT: 144.0 ± 0.7 Hz, KO: 144.6 ± 1.2 Hz; $P = 0.80$, $t_{(6)} = -0.42$; Cohen's $d = 0.38$, Student's *t*-test) between WT and KO mice. (**e**) Representative SPW-R in dCA1 triggered spectrograms averaged across entire representative sessions from WT and KO mice. (**f**) Averaged peak ripple amplitude was significantly lower in KO mice ($n = 4$ sessions) than in WT mice ($n = 4$ sessions). WT: 8.80 ± 0.24, KO: 8.00 ± 0.15; $P = 0.029$, $t_{(6)} = 2.84$, Cohen's $d = 2.32$, Student's *t*-test. (**g**) *Shank3*-KO mice generated fewer ripples with large amplitudes. Main effect of Genotype, $F_{(1,6)} = 6.24$, $P = 0.047$, $\eta^2 = 3.2\times10^{-5}$, partial $\eta^2 = 0.51$; Threshold, $F_{(1,6)} = 7958.7$, $P = 1.3\times10^{-10}$, $\eta^2 = 0.40$, partial $\eta^2 = 1.00$; Genotype×Threshold, $F_{(1,702)} = 7.74$, $P = 0.031$, $\eta^2 = 3.9\times10^{-4}$, partial $\eta^2 = 0.57$. *$P < 0.05$, **$P < 0.01$ by Tukey-Kramer multiple comparisons test. (**h**) Comparable online (left) and offline (right) activity similarity between pairs recorded in the dCA1 of WT ($n = 338$ pairs, 4 sessions) and KO ($n = 366$ pairs, 4 sessions) mice.

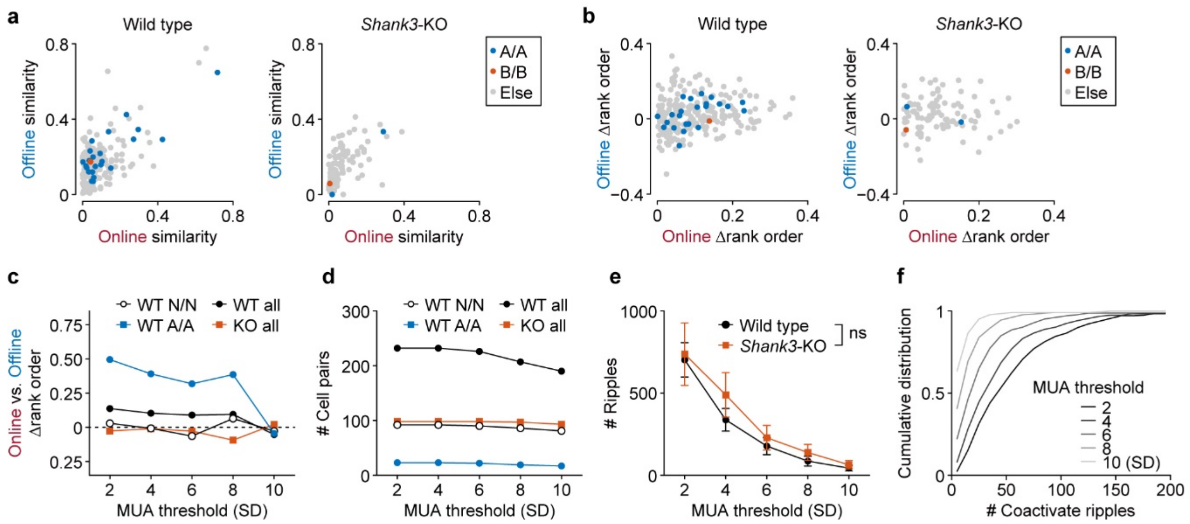

Supplementary Fig. 6 Additional analyses on the activity similarities and spike sequences.

(**a** and **b**) Scatter plots of the same data as in Fig. 5d (**a**; representing activity similarities between online theta cycles and offline ripples) or in Fig. 5k (**b**; representing delta rank orders within online theta cycles and offline ripples) but color-coded according to the social representation of each cell pair. Note the small proportion of mouse-B cell pairs in the data from WT mice and social neuron pairs in the data from KO mice. (**c** and **d**) Correlations of delta rank orders between online theta cycles and offline ripples (**c**) and the number of cell pairs having at least one coactive SPW-R (**d**) among four different subgroups (N/N cell pairs and A/A cell pairs in WT, all pyramidal cell pairs in WT or KO) upon varying the MUA threshold of SPW-Rs. (**e**) Number of SPW-Rs rapidly decreased upon varying the MUA threshold. ns, not significant by two-way repeated measures ANOVA. (**f**) Distribution of the number of coactive SPW-Rs across cell pairs including both genotypes.

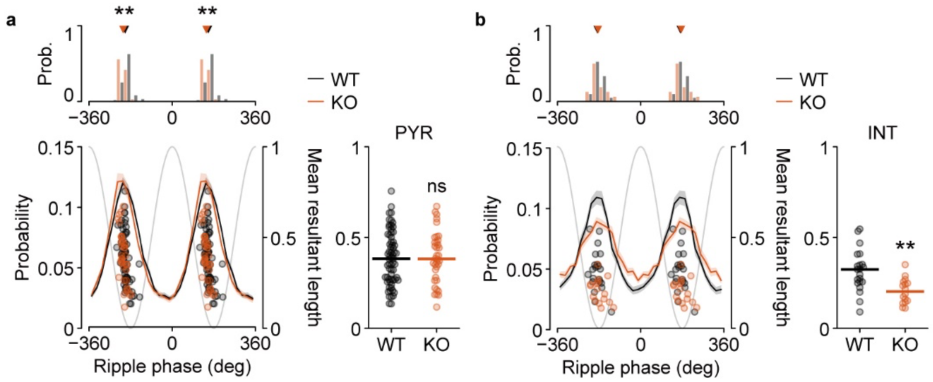

Supplementary Fig. 7 Spike-phase locking during offline ripples.

(**a** and **b**) Spike to ripple phase relationships of putative pyramidal cells (**a**; WT: *n* = 64 cells; KO: *n* = 36 cells) and interneurons (**b**; WT: *n* = 21 cells; KO: *n* = 16 cells). Note that only values from significantly (P < 0.001 by Rayleigh test) phase-locked neurons were plotted. Shaded areas represent SEMs. Black and red triangles on the histograms represent median values of the preferred ripple phases among cells from wild-type and Shank3-KO mice, respectively. ***P* = 0.0035, circular multi-sample median test. Dot plots show the comparison of the strength of ripple phase preference. ***P* = 0.0017, Wilcoxon rank sum test.
